# Effect of Phase Change Materials and Phase Change Temperature on Optimization of Design Parameters of Anaerobic Reactor Thermal Insulation Structure

**DOI:** 10.3390/ijerph19159020

**Published:** 2022-07-25

**Authors:** Feng Zhen, Yuwan Pang, Tao Xing, Hongqiong Zhang, Yonghua Xu, Wenzhe Li, Yong Sun

**Affiliations:** 1 Guangzhou Institute of Energy Conversion, Chinese Academy of Sciences, Guangzhou 510640, China; zhenfeng@ms.giec.ac.cn (F.Z.); xingtao@ms.giec.ac.cn (T.X.); 2 College of Engineering, Northeast Agricultural University, Harbin 150030, China; zhhqiong@163.com (H.Z.); funny021@163.com (Y.X.); 3 Guangdong Provincial Key Laboratory of New and Renewable Energy Research and Development, Guangzhou 510640, China; 4 Institute of Agricultural Resources and Environment, Guangdong Academy of Agricultural Sciences, Guangzhou 510640, China; 13580582341@139.com

**Keywords:** direct-absorption anaerobic reactor, glass envelope, design parameters, heat and mass transfer

## Abstract

Direct-absorption anaerobic reactors can maintain the fermentation process of microorganisms by utilizing solar absorption and scattering media in the biogas reactor to improve the slurry temperature. Direct-absorption heating alone can save the corresponding electric energy and ensure the normal fermentation process of the biogas slurry in the reactor, but there is still the problem of temperature fluctuation. In order to improve the stability of the fermentation process, it is proposed to optimize the design of this kind of reactor by adding paraffin phase change material. This article mainly studies the influence of paraffin phase change material added on the top and side of the reactor in the fermentation process and gives the corresponding design parameters for different climatic conditions, which lays a theoretical reference for the design process of this kind of reactor.

## 1. Introduction

Biogas production based on agricultural waste is a common method to solve resource and environmental issues, especially in a prominent agricultural country such as China [1]. The output of agricultural waste in rural areas of China is at an annual rate of 5~10%, which will increase to 50 billion tons by 2020 [2]. As an important model of sustainable agricultural development, biogas engineering is an important link to recycling and managing waste.

Biogas engineering can utilize the anaerobic fermentation of biomass to produce biogas by three temperature modes: low (15 °C), medium (30–38 °C), and high (50–55 °C) [3]. The efficiency of fermentation can be greatly affected by large temperature fluctuation [4]. Medium-temperature anaerobic fermentation has been shown to be more widely used owing to its better economy.

The research focus on biogas engineering is to produce clean energy via fermentation systems. For this aim, is propitious to improve the fermentation temperature and maintain biogas production efficiency under low-temperature conditions. Solar energy is an economical source for collecting heat, considering its universality of distribution. There are many heat-collecting technologies used in methane gas projects, including solar tubular collectors [5,6], solar air source heat pumps [7], and geothermal energy [8]. For instance, Vergil et al. [6] built a one-dimensional thermal computer simulation model to calculate the biogas slurry temperature on the basis of experimental validation and concluded that the slurry temperature was unable to reach 35 °C under solar heating. Most biogas heating systems cannot rely only on solar energy considering the problem of heating at night, though many systems have a regenerator. Multisystem heating modes, including a power-generation waste-heat-recovery system [9] and a biomass boiler [10] have been utilized to maintain the temperature in methane gas projects. Zhang et al. [10] used a solar energy heating system and a biogas boiler heating system to heat household digesters. Three groups of contrastive experiments were conducted to investigate the thermal performance of the heating system. The results showed that the biogas boiler can effectively supply the rest of the heat for the digesters.

The above-mentioned studies achieved the cooperative utilization of a variety of renewable energy sources and solved the problem of low production rate in biogas digesters under cold conditions. However, the systems have some defects, including complex pipelines, significant energy loss, and short periods of efficient utilization [7,8,9,10]. In comparison, the absorption methane system has the advantages of simple equipment and high energy utilization. The direct-absorption methane system can utilize solar absorption and scattering of media in the biogas digester to improve the temperature of the biogas slurry in order to maintain the fermentation process of microorganisms. The related studies in the field of solar photothermal utilization present experimental and theoretical analyses of salt gradient solar ponds [11]. Solar ponds are considered a good low-temperature heat source and are widely used for industrial purposes, such as the desalination of sea water [12,13]. Morteza et al. [13] utilized experimental and theoretical methods to investigate the heat transfer process in a salinity gradient solar pond and concluded that solar energy can effectively raise the water temperature and that water evaporation has a significant effect on the regenerative process. Previous studies have indicated that direct absorption of solar energy in medium-temperature anaerobic fermentation is feasible for heating the biogas slurry in the digester and that slurry evaporation is an important factor for its regenerative effect.

Compared with the brine in a solar pond, biogas slurry has small solid particles, including insoluble metabolites and substrate residue, which lead to a strong scattering effect for sunlight. The previous studies show that particle scattering has a significant effect on the regenerative process of direct-absorption solar thermal energy systems [14,15]. Bhalla et al. [14] investigated the effect of nanoparticles (Al_2_O_3_/Co_3_O_4_) on the photo-thermal process of a direct-absorption solar thermal collector and concluded that temperature increases in the collector when the media is filled with nanoparticles. So, the effect of particle scattering on the coupled heat and mass transfer process of a direct-absorption methane digester should be investigated.

Though the direct-absorption heating method alone can save the corresponding electric energy and ensure the normal fermentation process of the biogas slurry in the reactor, there is still a considerable problem of temperature fluctuation [16,17,18]. In order to improve the stability of the fermentation process it is proposed to optimize the design of this kind of reactor by adding paraffin phase change material [19,20]. In this article, the effects of paraffin phase change materials added on the top and side of the reactor in the fermentation process were studied, and the corresponding design parameters were given for different climatic conditions, which laid a theoretical reference for the design process of this kind of reactor.

## 2. Experimental Apparatus and Computational Model

### 2.1. Experimental Apparatus

**Figure 1:**
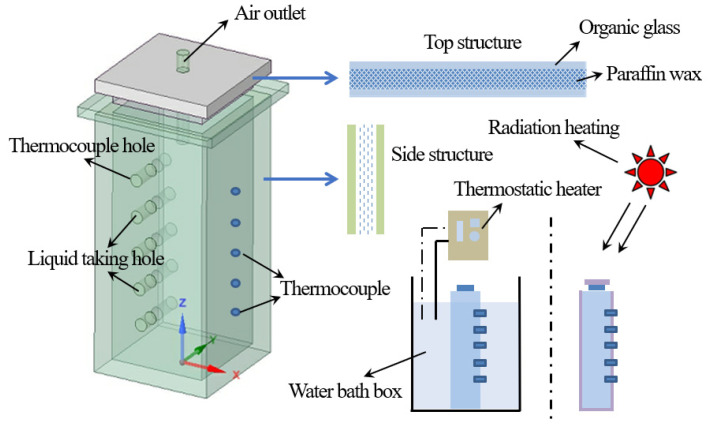
Structure drawing and component detail drawing of the direct-absorption anaerobic reactor.

The thickness of phase change material and other parameters greatly influence the solar energy absorption in a direct-absorption reactor. In this section, as shown in Figure 1 for the side and top insulation structure, different paraffin layer thicknesses and phase change temperatures are selected. Experimental and numerical calculation methods are used to analyze the insulation and heat storage effects of the insulation structure under various parameters, and to explore their impact on the fermentation process in the reactor. The formal experiment was carried out in Harbin from 12 July to 29 August 2019. Table 1 describes the parameters of the experimental equipment.

Three reactors were built to analyze the effect of solar irradiance on the fermentation process of different design parameters. As shown in Table 2, the ratios of C/N (carbon/nitrogen) for both groups of raw material are 25:1, which is suitable for gas production and concentration of ammonia nitrogen. The inoculum was obtained from a medium-temperature fermenter in the Research and Development Center of the Biomass Energy Engineering at Northeast Agricultural University, which uses cow dung as its raw material.

The C/N ratio for cow dung is 25:1 and the carbon content is 7.3%.

### 2.2. Computational Model

When the direct-absorption reactor works, the sunlight penetrates the thermal insulation structure and then enters the biogas slurry. Considering that the media involved are complex, varied, and contain particles, it is very difficult to directly solve the spectral transmission process of the whole system. In this paper, the absorption rate of the thermal insulation structure is first calculated, and then the status of the biogas liquid cavity is calculated by using its transmittance.

**Figure 2:**
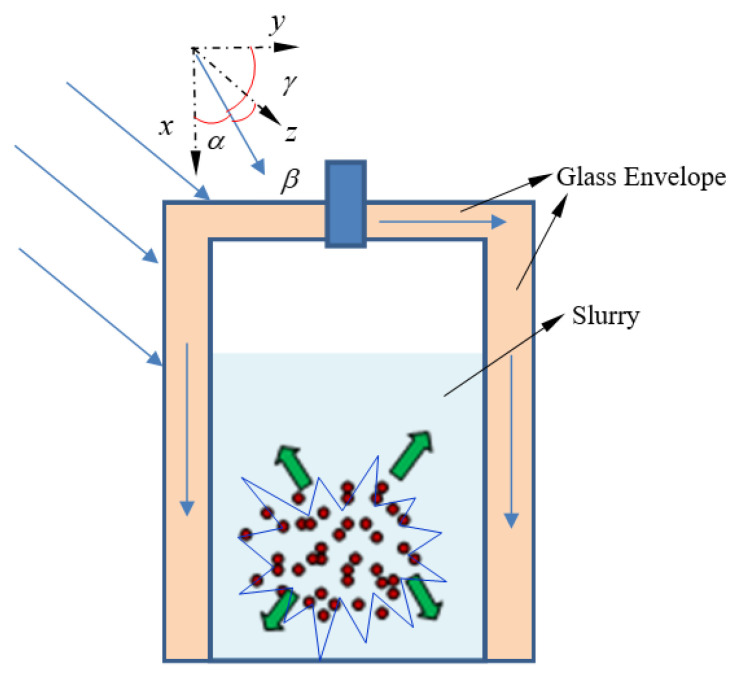
Principle of photothermal conversion in a direct-absorption anaerobic reactor.

As shown in Figure 2, glass, solid, and liquid paraffin are all pure absorbent media, regardless of their scattering effect on the sunlight. However, when calculating the spectral vector transmission process of the thermal insulation structure, the influence of the medium-temperature biogas slurry needs to be considered. Because the plexiglass material in the thermal insulation structure does not have strong spectral selectivity to sunlight, it is simplified as a grey body. The method of calculating the solar spectral absorption source term in the thermal insulation structure follows [21].

(1) $$\Phi (x,y,z)=I\left(I_{1},I_{2},I_{3},I_{4},\ldots ,I_{n}\right)\times \left[\begin{array}{l} \zeta_{11},\zeta_{12},\zeta_{13},\zeta_{14},\cdots \cdot \cdot ,\zeta_{1n}\\ \zeta_{21},\zeta_{22},\cdots \cdots \cdots \cdots ,\zeta_{2n}\\ \zeta_{31},\cdots \cdots \cdots \cdots \cdots \cdot ,\zeta_{3n}\\ \cdots \cdots \cdots \cdots \cdots \cdots \cdots \cdots \\ \cdots \cdots \cdots \cdots \cdots \cdots \cdots \cdots \\ \zeta_{n1},\zeta_{n2},\cdots \cdots \cdots \cdots ,\zeta_{nn} \end{array}\right]\times \left[\begin{array}{l} \cos\alpha_{1},\cos\beta_{1},\cos\gamma_{1}\\ \cos\alpha_{2},\cos\beta_{2},\cos\gamma_{2}\\ \ldots \ldots ,\ldots \ldots ,\ldots \ldots \\ \ldots \ldots ,\ldots \ldots ,\ldots \ldots \\ \cos\alpha_{n},\cos\beta_{n},\cos\gamma_{n} \end{array}\right]$$

where $I\left(I_{1},I_{2},I_{3},I_{4},\ldots ,I_{n}\right)$ represents the radiation intensity of each band of sunlight (W/(m^2^·μm·sr)), $\zeta_{ij}$ represents the spectral absorption function of each band and each region, and Formula (1) represents the cosine value of the angle between the sunlight and each direction, respectively.

**Figure 3:**
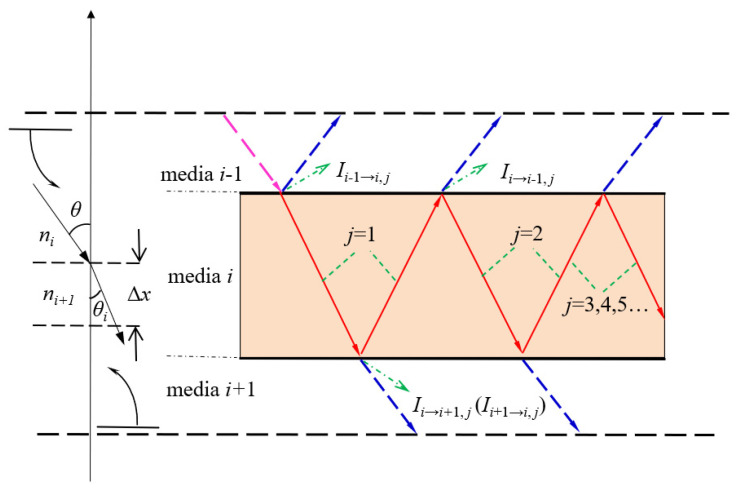
Local spectral vector transmission diagram of thermal insulation structure.

The spectral absorption function is needed to calculate the energy loss value of each stage according to the transmission path of sunlight, and the final statistical analysis can be carried out. As shown in Figure 3, due to the translucent structure of the whole direct-absorption biogas slurry fermentation system, considering the reflection effect of each surface, the sunlight will repeatedly shuttle through each layer. The specific radiation distance and path are related to the optical parameters of the medium, so the expression of the spectral absorption function is different for each region of the insulation structure. For example, if the outer glass of the top insulating layer is divided into *n*_1_ parts, the spectral absorption function of the first node is calculated as follows:

(2) $$\begin{array}{l} \zeta_{i1}=(1-\rho )\left(1-e^{-2k_{ti}b_{s}}\right)\{\left(1-e^{-k_{g}\frac{b_{1}}{2n_{1}}}\right)[1+e^{-2\left(k_{g}\left(b_{4}+b_{5}\right)+k_{si}b_{3b}+k_{li}b_{3a}\right)-k_{g}\left(b_{1}+b_{2}\right)-\frac{\left(2n_{1}-1\right)b_{1}}{2n_{1}}}\left(1-\rho_{g_{ou2}-l}\right)\\ \;\;\;\;\;\;\left(1-\rho_{l-s}\right)\left(1-\rho_{s-g_{in1}}\right)\left(1-\rho_{g_{in1}-a}\right)\left(1-\rho_{g_{in1}-s}\right)\left(1-\rho_{s-l}\right)\left(1-\rho_{l-g_{ou2}}\right)]+\\ \;\;\;\;\;\left(1-e^{-2k_{g}b_{1}}\right)e^{-\frac{4n_{1}-b_{1}}{2n_{1}}k_{g}}[\rho_{g_{ou2\;}-l}+e^{-2k_{li}b_{3a}}\left(1-\rho_{g_{ou2}-l}\right)(\rho_{l-s}+\left(1-\rho_{l-s}\right)\\ \;\;\;\;\rho_{s-g_{in1}}e^{-2k_{si}b_{3b}})]\}n_{i}^{2} \end{array}$$

(3) $$\rho_{i-j}=\frac{1}{2}\left[{\left(\frac{n_{i}\cos\varphi -n_{j}\cos\theta }{n_{i}\cos\varphi +n_{j}\cos\theta }\right)}^{2}+{\left(\frac{n_{j}\cos\varphi -n_{i}\cos\theta }{n_{j}\cos\varphi +n_{i}\cos\theta }\right)}^{2}\right]$$

## 3. Results and Discussion

The influence of PCM thickness in the side structure and top structure on the photochemical conversion process in the reactor is analyzed by experimental and numerical methods. The thicknesses of PCM for the two structures are 2, 4, and 6 cm, which represent I, II, and III, respectively.

### 3.1. Side Structure

**Figure 4:**
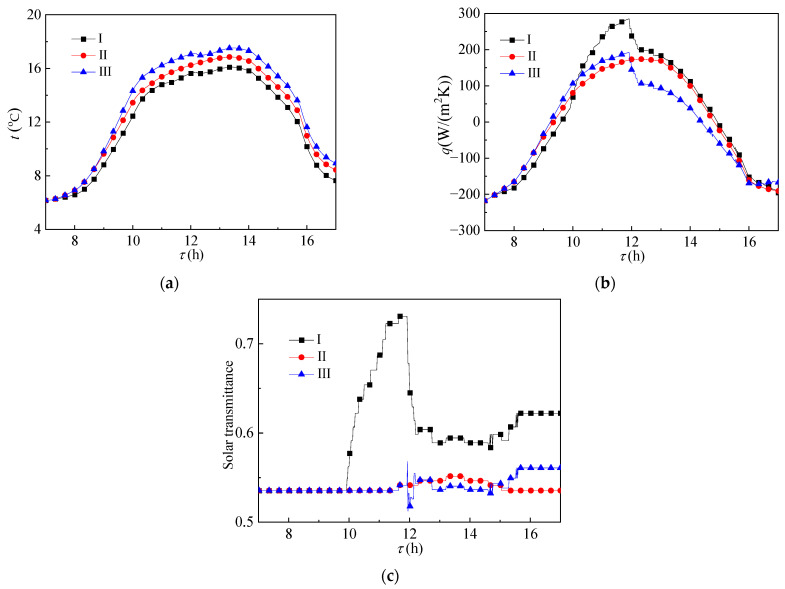
Change of (**a**) temperature, (**b**) heat flux, and (**c**) solar transmittance of the glass envelope over time.

Figure 4 shows the heat transfer effect of the glass envelope. As shown in Figure 4a, with the increase in phase change temperature, the temperature field inside the glass envelope gradually increases. The main reason is that, under the same environmental conditions, the paraffin material with low phase change temperature melts first and will absorb part of the solar radiation heat flow, resulting in lower temperature value. See Figure 4b,c for specific conditions. Figure 4b shows the change of heat flux and Figure 4c shows the change of solar transmittance. As the paraffin material filled in, the group I reactor melted first. The heat flow value was the lowest before 10:00, but its state was still dominated by solid state and the solar transmittance was similar to that of the other two groups. After most of its paraffin wax melted into liquid state, it stopped absorbing heat, the heat flux value was the highest, and the solar transmittance also increased to the highest value. Because the phase transition temperature of paraffin material filled in the group II reactor was closest to the ambient temperature, the change of heat flux in the group II reactor was the most uniform. The solar transmittance of the glass envelope of the group III reactor fluctuated around 12:00, mainly because its phase transition temperature was higher than the ambient temperature and would melt when the solar irradiation was strong and would solidify rapidly after melting, resulting in first an increase and then a decrease in solar transmittance. 

**Figure 5:**
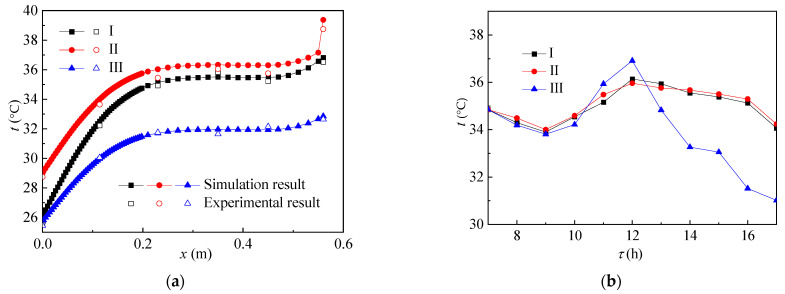
Temperature distribution with increase in (**a**) height and (**b**) time in the reactor.

**Figure 6:**
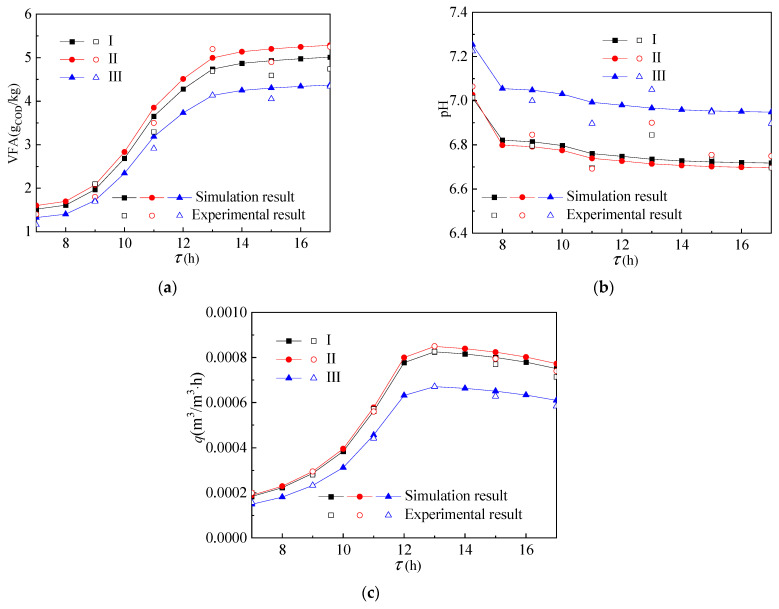
Change of (**a**) VFA, (**b**) pH, and (**c**) gas production of reactants over time.

Figure 5 shows the temperature distribution in the reactor. As shown in Figure 5a, the temperature value of the group II reactor was the largest, the temperature value of the group III reactor was the smallest, and the temperature value of the group I reactor was slightly lower than that of group II reactor. It shows that the phase transition temperature of the paraffin layer filled in the group II reactor was more in line with the actual environmental conditions. As shown in Figure 5b, the volume average temperature of the group II reactor was close to that of group I and its fluctuation was far less than that of the group III reactor. Moreover, the volume average temperature in the group III reactor decreased rapidly after 12:00. At the same time, as shown in Figure 6a–c, the acid and methane production of group I and group II were also larger than that of group III. The main reason is that the phase change temperature of the paraffin material used in the group III reactor was too high and it minimally melted in a one-day cycle, so it cannot have the effect of heat storage.

### 3.2. Top Structure

**Figure 7:**
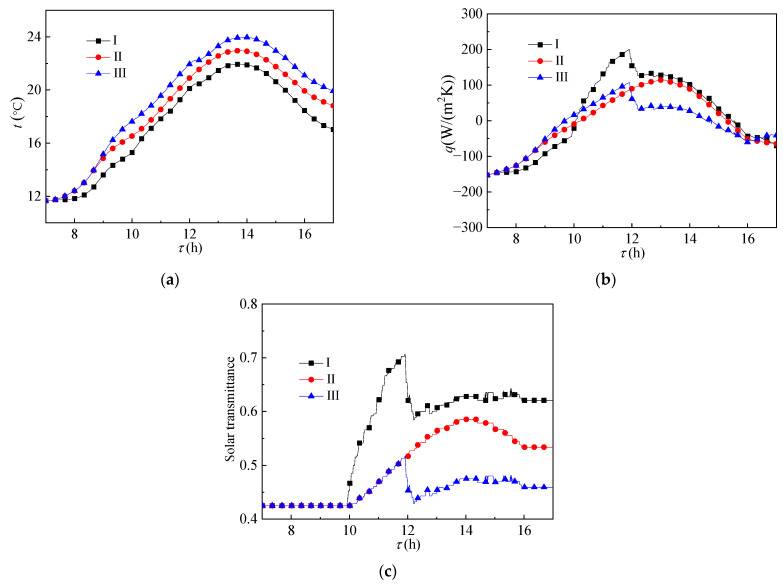
Change of (**a**) temperature, (**b**) heat flux, and (**c**) solar transmittance of the glass envelope over time.

Figure 7 shows the heat transfer effect of the glass envelope. As shown in Figure 7a, the same as for the side structure, the temperature value gradually increased with the increase in the phase transition temperature of the paraffin layer in the side structure. However, compared with the side structure, as shown in Figure 7b,c, due to the more effective solar irradiance received by the paraffin layer of the top structure, the paraffin layer of the group III reactor also began to melt at 12:00. Compared with the other two groups, its melting rate was lower, resulting in lower solar transmittance.

**Figure 8:**
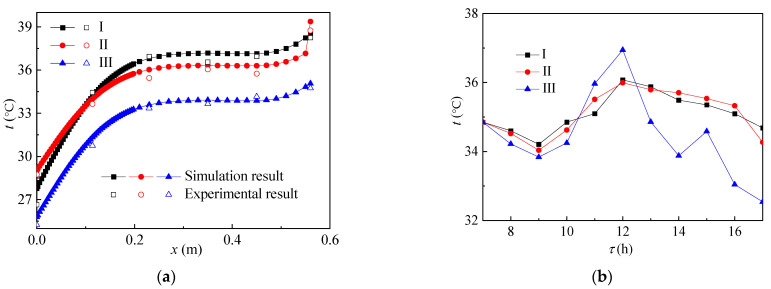
Temperature distribution with the increase in (**a**) height and (**b**) time in the reactor.

**Figure 9:**
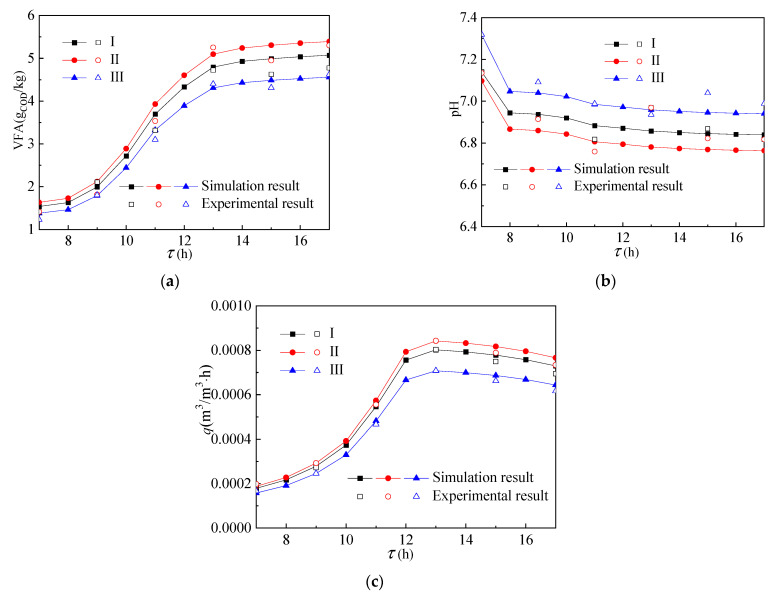
The change of (**a**) VFA, (**b**) pH, and (**c**) gas production of the reactants over time.

Figure 8 shows the temperature distribution in the reactor. As shown in Figure 8a,b, the variation trend of the temperature field and volume average temperature in the top structure with time was almost the same as that in the side structure. The temperature field and volume average temperature of group I and group II were larger than those of group III. The difference between the volume average temperature of the group III reactor and groups I and II is smaller than that of the side structure. As shown in Figure 9, the same situation also occurs in acid production and gas production. The main reason is that part of the paraffin in the group III reactor melted and had a certain heat storage effect.

## 4. Conclusions

Direct-absorption heating alone can save the corresponding electric energy and ensure the normal fermentation process of the biogas slurry in the reactor, but temperature fluctuation remains a significant problem. In order to improve the stability of the fermentation process, it is proposed to optimize the design of this kind of reactor by adding paraffin phase change material.

Filling the interlayer of the direct-absorption reactor with paraffin phase change material can effectively improve its thermal insulation and thermal storage performance, so as to improve its solar energy absorption efficiency and relative energy saving efficiency. The average volume temperature of the reactor decreased significantly in one day after adding paraffin material and the rate of acid and gas production was closer to that of the constant-temperature fermentation process. The phase change temperature of the paraffin layer in the side and top structures of the reactor has a great influence on its thermal insulation performance. With the change of phase transition temperature of the paraffin layer, the melting time changed from 10:00 to 12:00.

## Figures and Tables

**Table 1:** Parameter index of experimental instrument.

Number	Instrument	Range	Accuracy	Parameter
1	Total radiometer	1500 W/m^2^	5%	Total solar irradiation
2	Scattering radiometer	1500 W/m^2^	5%	Scattering solar irradiation
3	Radiant data collector	-	-	-
4	Thermocouple	−200–350 °C	1 °C	Slurry temperature
5	Thermometer	−50–50 °C	0.2 °C	Air temperature
6	Glass reactor	-	-	-
7	Air collecting bag	-	-	-
8	Insulation layer	-	-	-
9	Liquid intake	-	-	-
10	Computer	-	-	-

**Table 2:** Properties of raw materials for fermentation experiments.

Reactor	Raw Material	Volume	TS (Raw Material)	Weight (Raw Material) (g)	Weight (Raw Material) (g)	TS (Inoculum)	TS (Slurry)
A	Cow dung	11.5 L	24.37%	1862	9638	2.45%	6%
